# Bionomics of the primary arbovirus vectors *Aedes aegypti* and *Aedes albopictus* in southeastern Benin

**DOI:** 10.1186/s41182-025-00794-6

**Published:** 2025-08-22

**Authors:** Alphonse Keller Konkon, David Mahouton Zoungbédji, Rock Aïkpon, Isidore Hoyochi, Albert Sourou Salako, Camus Konkon, Antoine Salomon Lokossou, Brice Dangnon, Lamine Baba-Moussa, Martin Codjo Akogbéto, Germain Gil Padonou

**Affiliations:** 1 Centre de Recherche entomologique de Cotonou (CREC), Cotonou, 06 BP 2604 Benin; 2 Faculté des Sciences et Techniques de l, Université d’Abomey-Calavi, Abomey-Calavi, Benin; 3 École de gestion et d’exploitation des systèmes d’élevage, Université Nationale d’Agriculture de Porto-Novo, Porto-Novo, Benin; 4 Laboratory of Biology and Molecular Typing in Microbiology, Department of Biochemistry and Cellular Biology, Abomey-Calavi, Benin; 5 Ministère de la Santé, Cotonou, 08 BP 882 Benin; 6 Université Nationale des Sciences, Technologies, Ingénierie et Mathématiques (UNSTIM), Abomey, Benin; 7 Africa Youth Scientists Network, Cotonou, Benin

## Abstract

**Background:**

The main vectors of arboviruses, such as *Aedes aegypti* and *Aedes albopictus*, are present in Benin and deserve special attention in dengue prevention policies. In this context, the current study was initiated to provide information on the biology, ecology, including feeding behavior and life expectancy of *Aedes aegypti* and *Aedes albopictus* in southern Benin.

**Method:**

A larval survey was conducted in conjunction with a human landing catch (HLC), a Prokopak aspirator catch, and a survey of *Aedes* spp. breeding sites. The ovary dissection method was used to determine the age of the vectors. This allowed to assess the biology, ecology, exophagy or endophagy, and age expectancy of both *Aedes aegypti* and *Aedes albopictus* in southeastern Benin.

**Results:**

A total of 11 mosquito species were collected, with *Aedes aegypti* and *Aedes albopictus* showing the highest relative abundances, ranging, respectively, from 29.57% to 43.99% and from 16.26% to 45.65%, depending on the sampling method employed. Used tires accounted for 48.03% [45.06; 50.99] of all deposits found and were the most infested with *Aedes* spp. larvae, followed by buckets (14.23%) and jars (15.24%). The two main *Aedes* species studied (*Aedes aegypti* and *Aedes albopictus*) are more aggressive outdoors than indoors. Two peaks of aggressiveness were generally observed for both species: a first cycle in the morning from 7 a.m. to 11 a.m. and a second cycle in the evening from 4 p.m. to 7 p.m. A total of 76.47% of the *Aedes aegypti* and 81.21% of the *Aedes albopictus* samples were parous.

**Conclusion:**

Used tires and household containers (jars, water buckets, etc.) are the main breeding sites for *Aedes* spp., underscoring the importance of educating people about good water management habits. *Aedes aegypti* and *Aedes albopictus*, the main vectors of arboviruses, are exophagous and highly aggressive outdoors, with critical periods for human exposure, particularly in the morning and at the end of the day. Most of the collected females have already laid eggs (parous) and therefore are potentially susceptible to transmitting pathogens.

## Introduction

The genus of *Aedes* mosquitoes are insects from the arthropod phylum that have attracted medical attention for their involvement in the transmission of arboviruses [1]. *Aedes aegypti* and *Aedes albopictus* are the two main vectors within this genus that are widely implicated in arbovirus transmission cycles [2–4]. These species are recognized for their high vector competence, invasive behavior, and rapid ecological adaptability, enabling them to establish in diverse environments and contribute significantly to virus transmission dynamics [5, 6]. Native to Africa (*Ae. aegypti*) and Asia (*Ae. albopictus*), both species have now achieved nearly global distribution and frequently occur in sympatry across several regions [6, 7]. Their global expansion has been largely facilitated by increasing international trade, especially the movement of used tires and vehicles, alongside rapid urbanization and population growth over the past decades [8]. Among the various arboviral diseases transmitted by *Aedes* mosquitoes, dengue remains the most widespread and impactful [9, 10]. The disease has become increasingly concerning due to its emergence in new geographic areas [11–13]. The global epidemiological profile of dengue during the 2023–2024 period has been particularly alarming [14]. According to the World Health Organization (WHO), as of April 2024, over 7.6 million dengue cases had been reported globally. Notably, the number of cases recorded in the first four months of 2024 exceeded the total number of cases in 2023 by more than 4.6 million. During the same period, over 3000 dengue-related deaths were documented [14]. Africa accounted for the majority of these cases, with Burkina Faso alone representing 85% of the reported infections and 91% of the deaths. Additionally, other West African countries such as Senegal, Côte d’Ivoire, Nigeria, Ghana, and Mali have also experienced recurrent outbreaks of dengue, Zika, and chikungunya in recent years [15–20]. These findings highlight the considerable burden of arboviral diseases in West Africa and the persistent challenges in early diagnosis and effective surveillance systems across the region. In the absence of vaccines or antiviral treatments for most arboviruses, disease control efforts rely predominantly on vector control strategies [21, 22]. Thus, detailed information on vector biology, including ecological preferences, trophic behavior (anthropophagy or zoophagy), exophagic or endophagic tendencies, and lifespan, is essential to guide evidence-based interventions [23]. Both *Aedes aegypti* and *Aedes albopictus* have been involved in dengue outbreaks across multiple endemic and emerging areas [24, 25]. In addition to dengue, these species are also competent vectors for other medically important arboviruses such as Zika virus, chikungunya virus, and yellow fever virus [26]. Notably, their ecological and behavioral patterns may vary across different environments. In West Africa, and particularly in Benin, there is a scarcity of bionomic data on *Aedes* mosquitoes that transmit arboviruses. The present study was therefore designed to generate essential information on the biology, ecology, exophagic/endophagic behavior, and lifespan of *Aedes aegypti* and *Aedes albopictus* in southern Benin. These findings aim to inform and strengthen vector control programs through the development of context-specific strategies for the prevention and control of arboviral diseases in the country.

## Materials and methods

### Study sites

**Fig. 1:**
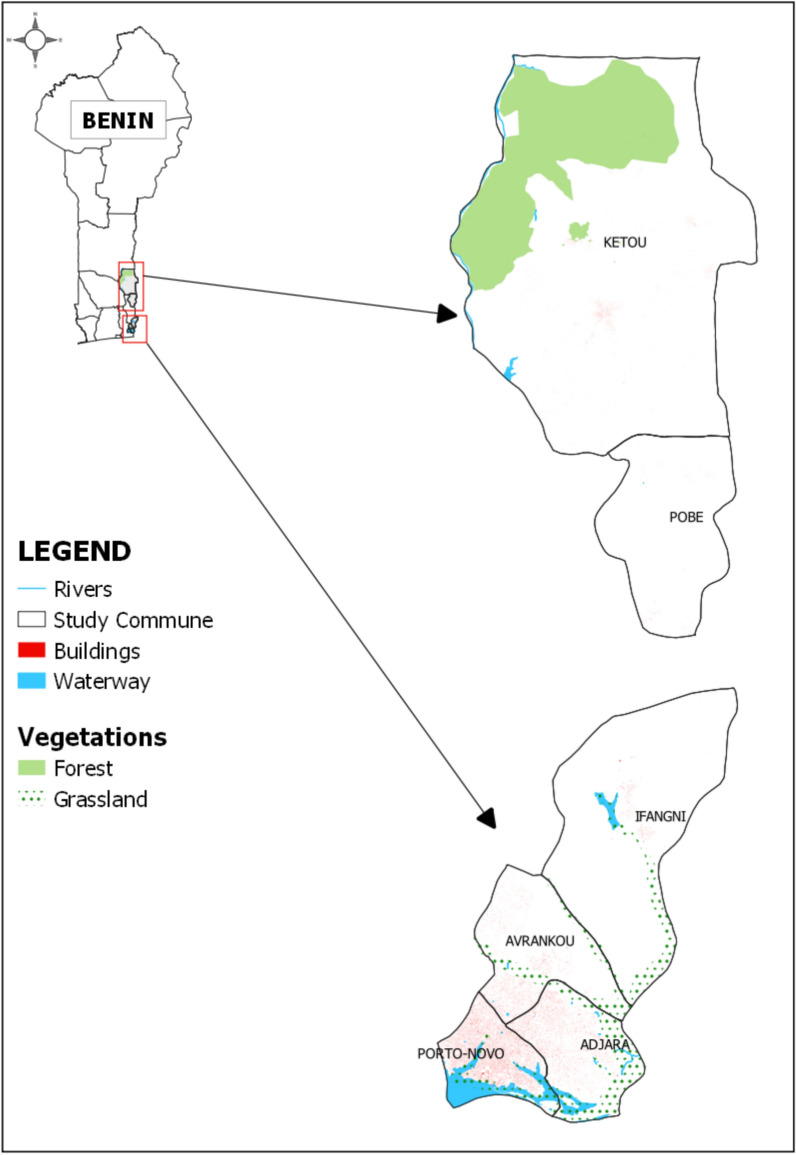
Study site

The study was conducted in six communities located in the southeastern region of Benin. The selected sites are Avrankou (6°33′00″ N, 2°40′00″ E), Adjara (6°32′00″ N, 2°40′00″ E), Pobè (6°58′00″ N, 2°41′00″ E), Ifangni (6°40′00″ N, 2°40′00″ E), Porto-Novo (6°29′50″ N, 2°36′18″ E), and Kétou (7°21′29″ N, 2°36′27″ E) (Fig. 1). These sites, which are all located in the departments of Ouémé and Plateau, were selected because of their favorable environmental characteristics for mosquito vectors.

### Entomological monitoring of arboviruses survey

The larval collection team is composed of two medical entomologists and a guide appointed by the district chief. Prior to larvae collection, the team has conducted a session of information awareness and obtained agreement from the community. After consent, all water containers inside and around each selected house were checked for larval presence. If a container is positive, a few samples of larvae/pupae are collected and preserved with the label of key information (date, house number, neighborhood name). Mosquito larvae collection was done in both districts in multiple *Aedes* mosquitoes breeding habitats including jars, canaries, cisterns, basins, barrels, tires, and abandoned carcasses. Collected larvae were transferred into containers and transported to the CREC insectarium for rearing until adult mosquitoes emerge. After emergence, all adults are identified morphologically using the keys of Edwards [27, 28]. The different breeding sites found are classified into several categories.

### Human landing catch

In each of the selected villages, two volunteer collectors are installed inside the household and two outside the household [29]. These volunteers were informed of the study objectives and provided informed consent. They will be medically screened and receive curative treatment in cases of illness. A total of eight collectors per village were trained prior to the start of the study. At each collection site, the collectors sat as bait, were barefoot and barelegged, and collected any mosquitoes that landed on their legs or feet before they bit in hemolysis tubes. Collections were made between 7 a.m. and 6 p.m. The first team of four collectors was replaced by another team of four collectors at 1 p.m. The collected *Aedes* spp. specimens were identified, referenced, stored on RNAlater, and grouped according to certain information, such as species, locality, date, and place of collection (indoor or outdoor). This technique was used to assess daily and hourly biting rates, as well as endophagic and exophagic trophic preferences.

### *Aedes* vectors sampling with Prokopack

Resting sites of Aedes mosquitoes were identified using the Prokopack aspirator, following the protocol described by Vazquez-Prokopec et al. [30]. This battery-powered, handheld device is specifically designed for the collection of resting mosquitoes both indoors and outdoors. Its portability and ease of use allow for efficient sampling within households, including under furniture (beds, wardrobes), behind curtains, on upper walls, on clothing, under dark objects, and on ceilings up to approximately 4 m in height. Prior to indoor collection, all doors and windows were closed to prevent mosquito escape. Sampling began in the innermost room and proceeded outward toward the front of the dwelling. Collections were conducted between 06:00 and 10:00, with an aspiration time of 10 to 15 minutes per room. In addition to indoor sampling, outdoor collections were also carried out in the immediate vicinity of each household. Potential resting sites such as large water barrels, discarded car tires, and other shaded structures were targeted. For each household, approximately 20 minutes were dedicated to outdoor aspiration of resting mosquitoes. Collected specimens were transferred into fine-mesh-covered, labeled paper cups for subsequent identification and laboratory analysis.

### Estimation of the physiological age of mosquitoes through ovarian dissection

The physiological age of female mosquitoes was assessed through ovarian examination using a standardized dissection technique under a stereomicroscope, followed by microscopic observation [31, 32]. A total of 30 *Aedes aegypti* and *Aedes albopictus* females per site were collected via human landing catches. Specimens were anesthetized by brief exposure to cold to preserve the integrity of internal tissues. Each mosquito was then placed in a drop of distilled water on a microscope slide and dissected using fine entomological needles. The ovaries were carefully extracted and examined under a compound microscope to estimate the physiological age based on the condition of the ovarian tracheoles. Mosquitoes were classified as either parous or nulliparous depending on whether the tracheoles appeared uncoiled (indicating prior oviposition) or tightly coiled (indicating no prior oviposition). The primary parameter derived from this analysis was the parity rate, which reflects the proportion of mosquitoes that had taken at least one blood meal and completed at least one gonotrophic cycle. Parous mosquitoes were considered physiologically older, while nulliparous individuals were classified as younger.

### Mosquito species identification

Morphological identification of collected mosquitoes was performed using the dichotomous keys of Huang [28] and Coetzee et al [33]. Aedes mosquitoes were carefully removed from their holding cages using a manual aspirator and transferred into transparent Petri dishes. To prevent escape during examination, specimens were immobilized by placing them in a −20 °C freezer for approximately five minutes. Subsequently, each mosquito was placed under a Leica EZ4E stereomicroscope for species and sex identification based on external morphological characteristics. Following identification, mosquitoes were individually preserved in 1.5-mL microcentrifuge tubes containing RNAlater^®^ solution, appropriately labeled, positioned upright in cryoboxes, and stored at −80 °C for downstream molecular analyses.

## Results

### Species diversity assessed through three mosquito sampling techniques

The diversity of mosquito species was investigated using three standard entomological collection methods commonly applied in medical entomology: human landing catches (HLC), larval and pupal surveys (larval prospection), and indoor resting collections using a Prokopack aspirator. A total of 11 mosquito species were identified, including five belonging to the genus Aedes: Aedes aegypti, Aedes albopictus, Aedes vittatus, Aedes africanus, and Aedes palpalis, with varying relative abundances depending on the sampling method. The proportion of Aedes aegypti ranged from 29.57% to 43.99%, while Aedes albopictus accounted for 16.26% to 45.65% of collected specimens. In addition to Aedes species, other mosquito genera were also recorded, including Culex nebulosus, Culex quinquefasciatus, Culex tigripes, Mansonia africana, Mansonia uniformis, and Anopheles gambiae (Table 1).

**Table 1:** Culicid species diversity according to different sampling methods: human landing catches (HLC), larval collection, and Prokopack aspiration

All area	HLC %(n)	Larval collection %(n)	Prokopack aspiration %(n)
*Aedes aegypti*	39,88(130)	43,99(490)	29,57(68)
*Aedes albopictus*	16,26(53)	33,33(378)	45,65(105)
*Aedes palpalis*	18,71(61)	0,00(0)	7,39(17)
*Aedes africanus*	0,92(3)	0,00(0)	0,00(0)
*Aedes vittatus*	2,45(8)	0,00(0)	0,00(0)
*Culex quinquefasciatus*	19,02(62)	17,15(191)	15,22(35)
*Culex nebulosus*	0,31(1)	3,77(42)	0,00(0)
*Culex tigripes*	0,00(0)	1,17(13)	0,00(0)
*Mansonia africana*	0,92(3)	0,00(0)	0,00(0)
*Mansonia uniformis*	0,31(1)	0,00(0)	0,00(0)
*Anopheles gambiae sl*	1,23(4)	0,00(0)	2,17(5)
**Total**	**326**	**1114**	**230**

### Characteristics of different Aedes breeding sites

A total of 1089 breeding sites were inspected, with used tires being the most common and most infested with *Aedes* spp. larvae, accounting for 48.03% [45.06; 50.99] and 67.99% [64.01; 71.97] of all sites found with *Aedes* spp. larvae, respectively. Buckets (14.23%) and glasses (15.24%) were also representative in terms of numbers. In addition, other categories of roosting sites were present, although in smaller numbers than the previous ones: barrels (7.99%), cans (1.61%), drinking troughs (1.93%), tree holes (2.57%), flower pots (1.74%), and abandoned vehicles (1.65%). In addition, these sites accounted for 5.11%, 4.36%, 0.57%, 1.70%, 1.52%, and 1.33% of all positive sites (n=528), respectively (Table 2).

**Table 2:** Characteristics of different *Aedes* breeding sites

Name of container	Number of container	% Container IC 95 %	Number of positive container	% positive Container IC 95 %	Characteristics
Buckets	155	14,23[12,16 ; 16,31]	70	13,26[10,36 ; 16,15]	Exposed (not covered)
Jars	166	15,24[13,11 ; 17,38]	22	4,17[2,46 ; 5,87]	Domestic water storage
Used tires	523	48,03[45,06 ; 50,99]	359	67,99[64,01 ; 71,97]	Isolated
Cars	18	1,65[0,90 ; 2,41]	7	1,33[0,35 ; 2,30]	Discarded, stagnant water
Discarded containers	72	1,61[5,15 ; 8,09]	23	4,36[2,61 ; 6,10]	Waste disposal, exposed
Tanks	87	7,99[6,38;9,60]	27	5,11[3,23 ; 6,99]	Water storage
Drinking troughs	21	1,93[1,11 ; 2,75]	3	0,57[-0,07 ; 1,21]	Domestic, daily use
Tree holes	28	2,57[1,63;3,51]	9	1,70[0,60 ; 2,81]	Natural
Flower pot	19	1,74[0,97;2,52]	8	1,52[0,47 ; 2,56]	Humid, shady, decorative
N total	1089		528		

*N* : number, *CI*: confidence interval

### Hourly human biting rate of *Ae. aegypti* and *Ae. albopictus* in the departments of Ouémé and Plateau

**Fig. 2:**
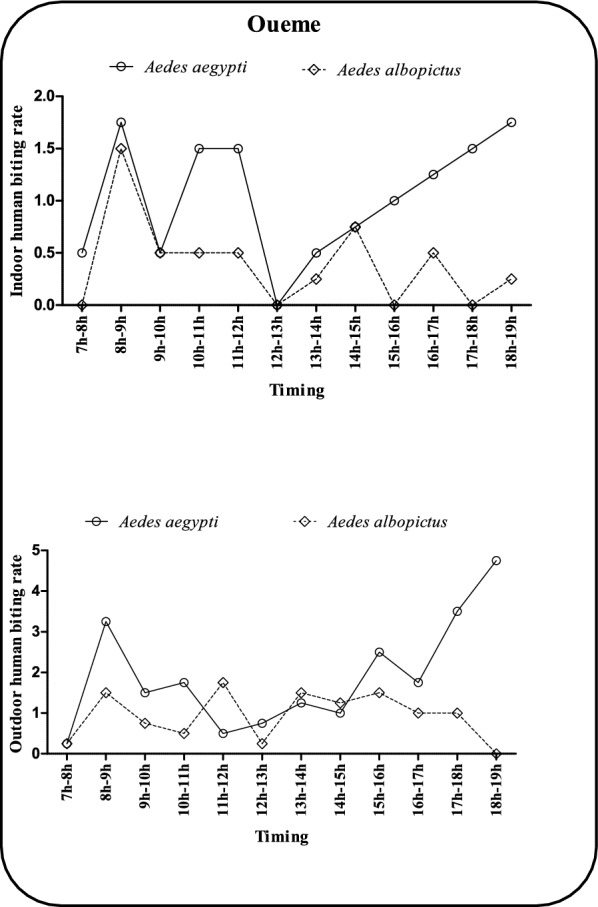
Hourly variation in human biting rate of *Ae. aegypti* and *Ae. albopictus* in the Oueme region

**Fig. 3:**
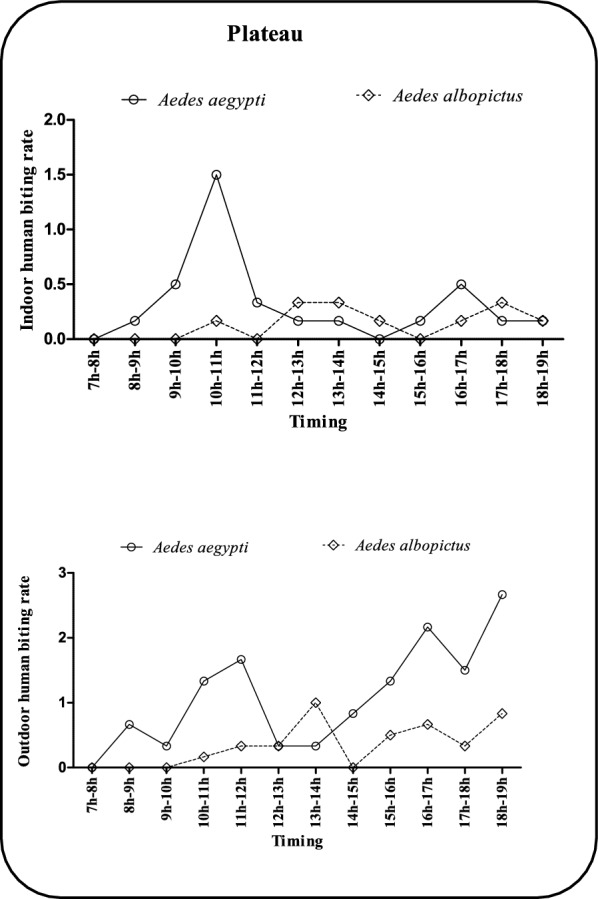
Hourly variation in human biting rate of *Ae. aegypti* and *Ae. albopictus* in the Plateau region

Figures 2 and 3 show the hourly human biting rate cycles of the two main dengue vector species in two departments of Benin, namely, Ouémé (municipalities of Avrankou, Adjarra and Porto-Novo) and Plateau (Ifangni, Pobè and Kétou). In Ouémé (Fig. 2), *Ae. aegypti* shows three periods of aggressiveness inside dwellings. The first peak from 7 am to 9 am exceeded 1.5 bites/man/hour, and the second peak was 1.5 bites/man/hour between 9 am and 12 noon. A third cycle of aggressiveness exceeding 1.5 bites/man/hour is observed between 3:00 pm and 7:00 pm. In addition to these highly aggressive hours, the bite rate varies between 0 and 0.5 bites/man/hour for the remainder of the day. For *Ae. albopictus*, a first peak of aggressiveness of 1.5 bites/man/hour was observed between 7 am and 10 am. Two further peaks occurred between 14:00 and 15:00 and between 16:00 and 18:00, with hourly bite rates hovering at approximately 0.5 bites/hour. Outdoors, *Ae. aegypti* also exhibits three periods of high aggressiveness. The first is from 7 a.m. to 9 a.m., when more than 3 bites/hour are recorded, followed by a second period from 3 p.m. to 4 p.m., when biting rate exceeds 2 bites/hour. The last period is from 17:00 to 19:00, with an aggressiveness of up to 5 bites/hour. For *Aedes albopictus*, aggressiveness varied between 0 and 1.5 bites/man/hour throughout the day. In the plateau region (Fig. 3), two peaks of aggressiveness were observed indoors. A peak in aggressiveness of 1.5 bites/man/hour was observed. A second peak in aggressiveness was observed between 4 pm and 5 pm. Among these periods of high aggressiveness, the other periods were characterized by a variation in bite rate from 0 to 0.5 bites/man/hour. On the other hand, the indoor aggressiveness of *Ae. albopictus* fluctuated between 0 and 0.5 bites/man/hour throughout the day. The outdoor aggressiveness of *Ae. aegypti* showed three peaks in the plateau area. A peak of 2 bites/hour is observed between 10 and 12 a.m., followed by a second peak between 4 and 5 p.m., when aggressiveness exceeds 2 bites/hour. Finally, a third peak of 3 bites/person/hour was observed between 6 pm and 7 pm. In addition, a single peak of aggressiveness of 1 bite/person/hour was observed in *Ae. albopictus* between 1 pm and 2 pm. All other periods of the day were characterized by aggressiveness ranging from 0 to less than 1 bite/person/hour.

### Determination of the resting places of the main dengue vectors

Table 3 shows the endophilia and exophilia rates of the main dengue vector species in the six communities of Benin. Endophilia rates range from 0% to 38.46% for *Ae. aegypti*, whereas exophilia rates range from 0% to 100%. On average, the exophilia rate (76.47%) of the *Ae. aegypti* collected from all the communities was much higher than the endophilia rate (23.53%). Endophilia rates varied from 6.45% to 30.49% in *Ae. albopictus*, whereas exophilia rates ranged from 69.51% to 100%. In addition, 81.77% of the *Ae. albopictus* were exophilic, whereas 18.23% were endophilic. In general, the two main vector species (*Ae. aegypti* and *Ae. albopictus*) are more exophilic than endophilic.

**Table 3:** Resting places of *Aedes* vectors

Communes	Species	Total	Endophilic %(n)	Exophilic %(n)		p-value
Avrankou	*Aedes aegypti*	0	0 (0)	0 (0)		–
	*Aedes albopictus*	82	30,49 (25)	69,51 (57)		< 0,0001
Adjara	*Aedes aegypti*	2	0 (0)	100 (2)		0,3173
	*Aedes albopictus*	31	6,45 (2)	93,55 (29)		< 0,0001
Pobe	*Aedes aegypti*	16	6,25 (1)	93,75 (15)		< 0,0001
	*Aedes albopictus*	14	7,14 (1)	92,86(13)		< 0,0001
Ifangni	*Aedes aegypti*	4	0(0)	100(4)		0,0339
	*Aedes albopictus*	19	5,26 (1)	94,74 (18)		< 0,0001
Porto-Novo	*Aedes aegypti*	39	38,46 (15)	61,54 (24)		0,07
	*Aedes albopictus*	18	22,22 (4)	77,78 (14)		0,0027
Kétou	*Aedes aegypti*	7	0 (0)	100 (7)		0,0013
	*Aedes albopictus*	17	0 (0)	100 (17)		< 0,0001
Total	*Aedes aegypti*	68	23,53 (16)	76,47 (52)		< 0,0001
	*Aedes albopictus*	181	18,23 (33)	81,77(148)		< 0,0001

%: percentage, *n*: number

### Estimation of the physiological age of *Ae. albopictus* and *Ae. aegypti*

A total of 352 *Aedes* ovaries were dissected for the longevity study (Table 4). In fact, 187 *Ae. aegypti* and 165 *Ae. albopictus* individuals collected from human volunteers were dissected. The paturity rate ranged from 52.38% [36.42–68] to 90% [73.47–97.89], with an average of 76.47% [69.73–82.35] for *Ae. aegypti*. In *Ae. albopictus*, it ranged from 70% [50.6–85.27] to 100% [88.43–100], with an average of 81.21% [74.4–86.86]. These results show that more than 75% of both vector populations (*Ae. aegypti* and *Ae. albopictus*) had at least one blood meal, laid at least once, and were therefore older. However, no significant difference was detected between the feeding rates of the two vector species considered in this study, neither in each of the communities nor in the accumulation of all the communities.

**Table 4:** Estimation of the physiological age of Ae. albopictus and Ae. aegypti

Communes	Species	N_ dissected	N_parous	Parous rate (%)	CI 95%
Avrankou	*Aedes aegypti*	30	27	90,00	[47–97]
	*Aedes albopictus*	30	30	100,00	[43–100]
Adjara	*Aedes aegypti*	30	26	86,67	[24, 28–96]
	*Aedes albopictus*	25	18	72,00	[50,61–87,93]
Pobe	*Aedes aegypti*	42	22	52,38	[36, 42–68]
	*Aedes albopictus*	30	21	70,00	[6–85]
Ifangni	*Aedes aegypti*	30	24	80,00	[29, 43–92]
	*Aedes albopictus*	30	22	73,33	[11–87]
Porto-Novo	*Aedes aegypti*	30	23	76,67	[57,72–90,07]
	*Aedes albopictus*	30	27	90,00	[47–97]
Kétou	*Aedes aegypti*	25	21	84,00	[63, 92–95, 46]
	*Aedes albopictus*	20	16	80,00	[27, 34–94]
Total	*Aedes aegypti*	187	143	76,47	[69, 73–82, 35]
	*Aedes albopictus*	165	134	81,21	[4–86]

*N*: number, *CI*: confidence interval

## Discussion

This study provides comprehensive data on the biology, ecology, exophagic/endophagic behavior, and life cycle of *Aedes aegypti* and *Aedes albopictus* in southern Benin. These findings serve as a foundation for vector control programs to develop effective strategies for the prevention and management of arboviral diseases. The results on mosquito species diversity revealed a total of eleven mosquito species, including five belonging to the genus *Aedes*. This finding reflects a high level of species richness in southern Benin, where the study was conducted. *Aedes aegypti* and *Aedes albopictus* were the predominant species, with relative abundances ranging from 29.57% to 43.99% and from 16.26% to 45.65%, respectively. These results highlight the ability of these vectors to thrive in domestic and peri-domestic environments, as well as their potential epidemiological significance as arbovirus vectors [34]. *Aedes aegypti* and *Aedes albopictus* were more frequently collected through human landing catches (HLC) and larval surveys, confirming their anthropophilic behavior and preference for artificial breeding sites [35]. Moreover, the presence of *Anopheles gambiae*, the principal malaria vector, although recorded at low density in this study, indicates the potential coexistence of malaria and arboviral transmission risks within the same geographical area [36, 37]. The assessment of breeding habitat typology revealed that discarded used tires serve as the most preferred and ecologically suitable larval development sites for Aedes mosquitoes. These were the most frequently encountered and heavily infested larval habitats. Barrels and clay jars represented the second most common and infested breeding containers. Given that Benin is a major importer of used tires from Europe, these materials often become environmentally abandoned after a short period of use [34, 38, 39]. This situation explains the high prevalence of tire-associated larval habitats observed in the present study and corroborates findings reported across Africa and other regions [40–42]. Furthermore, tires provide a microclimate favorable to *Aedes* proliferation, characterized by low light exposure and high humidity [1, 6]. Previous research suggests that *Aedes albopictus* was introduced into continental Africa through the trade of used tires [43]. Additionally, the use of clay jars for storing fresh water is a long-standing cultural practice in many African communities [44]. This traditional behavior persists over time, which explains the significant number of jars recorded in this study [44]. These containers are commonly used for household water storage [45]. The evaluation of host-seeking behavior of *Ae. aegypti* and *Ae. albopictus* revealed that both species are predominantly exophagic. In this study, 76.47% of *Ae. aegypti* and 81.77% of *Ae. albopictus* were collected outdoors using a Prokopack aspirator. These results are consistent with those of Lounibos et al. [46], who reported a high degree of exophily in both species. The observed exophilic behavior of *Ae. aegypti* in this region contrasts with its endophagic and endophilic tendencies in Asia and Latin America, where it typically feeds and rests indoors. In contrast, *Ae. albopictus* is known to be opportunistically exophagic and exophilic [47, 48].In West Africa, unlike observations from Côte d'Ivoire that suggest endophilic and endophagic behaviors [38, 49], a recent study conducted in Ghana reported that 76% of *Ae. aegypti* specimens were collected outdoors, while only 24% were captured indoors [46], supporting our findings. Similar trends were reported on Réunion Island (France, Indian Ocean), where [20] estimated that 89% of *Ae. aegypti* were exophagic. The predominantly exophagic behavior of these vectors suggests that indoor vector control interventions such as long-lasting insecticidal nets (LLINs) and indoor residual spraying (IRS) may be less effective. This underscores the need to prioritize outdoor-based control strategies in the event of arbovirus outbreaks [50–52]. Our findings also identified three distinct peaks of aggressive biting activity for both vector species throughout the day: the first occurring between 7:00 and 9:00 AM (sometimes extending to 10:00 AM), the second between 10:00 AM and 12:00 PM (occasionally extending to 1:00 PM), and the third between 4:00 and 7:00 PM. [14] reported comparable biting periods in Sri Lanka, from 5:00 to 11:00 AM and 3:00 to 7:00 PM, which are consistent with our observations. Moreover, our study confirms the exophilic resting behavior of both *Ae. aegypti* and *Ae. albopictus*. Other investigations have shown that *Ae. aegypti* may rest indoors in bedrooms, kitchens, and closets, whereas *Ae. albopictus* prefers peri-domestic vegetated and shaded environments [53, 54]. A recent study conducted in Kinshasa, Central Africa, further corroborated these findings, indicating that both species exhibit exophilic tendencies [55]. The study also revealed that a significant proportion of *Aedes* vectors (*Ae. albopictus*: 81.21%; *Ae. aegypti*: 76.47%) were parous females, having completed at least one gonotrophic cycle. Although the method employed does not provide precise age estimation, it confirms that the vectors had oviposited at least once. Although the study was geographically limited and conducted over a restricted timeframe, the results provide critical insights for improving vector surveillance, personal protection strategies, and targeted vector control interventions.

## Conclusion

This study provides data on the biology, ecology, exophagy/endophagy, and life expectancy of *Aedes aegypti* and *Aedes albopictus* in southeastern Benin. *Aedes* spp. breed in jars and barrels used for domestic purposes and in used tires left uncovered in the environment. The exophagous nature of the two main vectors has been observed. *Aedes aegypti* and *Aedes albopictus*, the main vectors of arboviruses, are exophagous and characterized by high outdoor aggressiveness, with critical periods for human exposure, particularly in the morning and at the end of the day. Most of the vectors collected were elderly (parous) and therefore potentially competent for transmission. The present study therefore serves as a basis for future epidemiological surveillance in the country.

## Data Availability

The datasets that were analyzed in this study are available from the corresponding author and the lead author.

## Abbreviations

CREC: Centre de recherche entomologique de Cotonou
WHO: World Health Organization
LLINs: Long-lasting insecticidal nets
IRS: Indoor residual spraying
HLC: Human landing catches
